# Longitudinal Study of Informed Consent in Innovative Therapy Research: Experience and Provisional Recommendations from a Multicenter Trial of Intracerebral Grafting

**DOI:** 10.1371/journal.pone.0128209

**Published:** 2015-05-26

**Authors:** Laurent Cleret de Langavant, Sophie Sudraud, Christophe Verny, Pierre Krystkowiak, Clémence Simonin, Philippe Damier, Jean-François Demonet, Frédéric Supiot, Amandine Rialland, David Schmitz, Patrick Maison, Katia Youssov, Anne-Catherine Bachoud-Lévi

**Affiliations:** 1 AP-HP, Hôpital Henri Mondor, Centre de Référence—Maladie de Huntington, Neurologie cognitive, Créteil, France; 2 Université Paris Est, Faculté de médecine, Créteil, France; 3 INSERM U955, Equipe 01, Neuropsychologie Interventionnelle, Créteil, France; 4 Département d’Etudes Cognitives, Ecole Normale Supérieure, PSL Research University, Paris, France; 5 CHU d'Angers, Centre de Référence des Maladies Neurogénétiques, service de neurologie, Angers, France; 6 UMR CNRS 6214 INSERM U1083, CHU d'Angers, Angers, France; 7 CHU d’Amiens, service de neurologie, Amiens, France; 8 EA 4559, Laboratoire de Neurosciences Fonctionnelles et Pathologies, Université de Picardie Jules Verne, Amiens, France; 9 Université Lille Nord de France, Lille, France; 10 INSERM UMR837, Institut de Recherches sur le Cancer de Lille (IRCL), Lille, France; 11 CHRU Lille, Département de Neurologie et des Mouvements Anormaux, Lille, France; 12 CHU Nantes, Service de Neurologie, Nantes, France; 13 UFR Médecine, Université de Nantes, Nantes, France; 14 Leenaards Memory Centre, Clinical Neuroscience Department, Lausanne University Hospital (CHUV), Lausanne, Switzerland; 15 INSERM U825, Université de Toulouse, Toulouse, France; 16 Cliniques Universitaires de Bruxelles Hôpital Erasme, Bruxelles, Belgique; 17 AP-HP, Hôpital Henri Mondor, Unité de Recherche Clinique, Creteil, France; Inserm U837, FRANCE

## Abstract

**Background:**

There is an urgent need to assess and improve the consent process in clinical trials of innovative therapies for neurodegenerative disorders.

**Methods:**

We performed a longitudinal study of the consent of Huntington’s disease patients during the Multicenter Fetal Cell Intracerebral Grafting Trial in Huntington’s Disease (MIG-HD) in France and Belgium. Patients and their proxies completed a consent questionnaire at inclusion, before signing the consent form and after one year of follow-up, before randomization and transplantation. The questionnaire explored understanding of the protocol, satisfaction with the information delivered, reasons for participating in the trial and expectations regarding the transplant. Forty-six Huntington’s disease patients and 27 proxies completed the questionnaire at inclusion, and 27 Huntington’s disease patients and 16 proxies one year later.

**Results:**

The comprehension score was high and similar for Huntington’s disease patients and proxies at inclusion (72.6% vs 77.8%; *P* > 0.1) but only decreased in HD patients after one year. The information satisfaction score was high (73.5% vs 66.5%; *P* > 0.1) and correlated with understanding in both patients and proxies. The motivation and expectation profiles were similar in patients and proxies and remained unchanged after one year.

**Conclusions:**

Cognitively impaired patients with Huntington’s disease were capable of consenting to participation in this trial. This consent procedure has presumably strengthened their understanding and should be proposed before signing the consent form in future gene or cell therapy trials for neurodegenerative disorders. Because of the potential cognitive decline, proxies should be designated as provisional surrogate decision-makers, even in competent patients.

## Introduction

Informed consent is a prerequisite for research on humans worldwide, but it is particularly challenging to obtain meaningful consent in studies of highly innovative therapies, such as intracerebral cell grafting or gene therapy for neurodegenerative diseases. Indeed, the principle that consent requires a free, voluntary and informed decision by a competent person can be at odds with the declining competence of patients with neurodegenerative diseases [1–3]. Furthermore, consent is a particularly important and difficult issue in intracerebral cell transplantation trials, due to the very nature of these trials. First, the benefit/risk balance of such innovative treatments is difficult to evaluate and cannot be predicted from animal studies [4, 5]. Second, intracerebral grafts cannot subsequently be removed and their effects may persist after the trial has been completed. Third, therapeutic misconception—a failure to understand that the defining purpose of clinical research is to produce generalizable knowledge [6]—may be particularly frequent in the setting of cell therapy [7]. Presumably, it is because these therapies target severe neurodegenerative diseases that the idea of regeneration often generates dramatic hopes [8]. Despite these unresolved issues, cell transplantation trials are already underway for Parkinson’s disease (PD) and Huntington’s disease (HD) [9–12]. Here, we examined the consent procedure in an ongoing cell transplantation trial, to determine the roles of patients and proxies in the consent process, with a view to optimizing this process for future trials evaluating innovative therapies in neurodegenerative disorders.

It is tempting to turn to proxies as surrogate decision-makers when trying to obtain informed consent for the inclusion of a patient in a clinical trial, as is often the case in dementia [1], schizophrenia [13], and pediatric studies [14]. However, depriving patients of the possibility of providing consent goes against the principle of individual autonomy [15]. The International Society for Stem Cell Research (ISSCR) task force recommends that “research subjects’ comprehension of relevant information—especially of the risks and uncertainties—be evaluated at the time of obtaining consent” [16], but does not specify how this comprehension should be assessed. In a recent study in which the consent of patients with Parkinson’s disease (PD) for participation in drug or gene therapy trials was simulated, a psychometric cutoff was proposed, to identify patients at risk of having an impaired capacity to consent; the authors concluded that these patients should be evaluated through a structured assessment of capacity and that a study partner should be designated [2].

In this study, we made use of the consent data from the Multicenter Intracerebral Grafting in Huntington’s Disease trial (MIG-HD). We prospectively investigated whether the HD patients were able to provide valid consent and the role of proxies as potential surrogate decision-makers. The MIG-HD study provided us with a unique opportunity to refine consent procedures for future cell or gene therapy trials on the basis of real, rather than simulated data. Many features of HD make it a good model for consent in neurodegenerative disorders. It is an incurable inherited disease that manifests in adulthood with motor, cognitive and behavioral disorders and leads to death within about two decades. The severity of HD and the current lack of curative treatment may affect the decisions taken by patients, leaving them in a position of susceptibility to coercion. Unreasonable hope or the blind trust patients have in their doctors may also influence their choices [15, 17]. Critically ill patients may be more motivated than less severely ill patients with alternative treatments open to them by the prospect of personal benefit than by altruism and making a contribution to scientific knowledge [17]. Therapeutic misconception may, therefore, be particularly frequent among HD patients participating in therapeutic research. In addition, the executive dysfunction, memory deficits and unawareness of symptoms of HD patients may lead to impairments of understanding, information recall, and decision-making relating to the research [18, 19]. Finally, as HD patients display a gradual cognitive decline, the stability of their consent once enrolled in a therapeutic trial can be called into question [20, 21].

Given the length and complexity of the MIG-HD study and the patients’ vulnerability, a consent questionnaire was designed, to ascertain whether the patients were able to give free and informed consent, and to determine whether the views of their proxies were similar to their own. We studied the responses given by patients and their proxies, to improve the consent procedure for patients with neurodegenerative disorders participating in clinical trials for innovative therapies.

## Methods

The ongoing MIG-HD study (ClinicalTrials.gov identifier: NCT00190450) is assessing human fetal cell allografts in patients with early-stage HD at six centers in France and Belgium. The grafts are implanted stereotactically and bilaterally, in the caudate and putamen. Behavioral, cognitive, psychiatric, biological, video, electrophysiological and neuroimaging data are collected throughout the study. The MIG-HD study has been approved by the Research Ethics Committee of Henri Mondor Hospital (Créteil, France). All participants in this study gave their written informed consent.

### Participants and the informed consent procedure

Fifty-four patients with early-stage HD (total functional capacity score [22] ≥ 10; MDRS [23] > 120) have been enrolled in the MIG-HD trial for a duration of 52 months for each patient. The designation of a proxy is mandatory for participation. Investigators provide patients and proxies with oral information and a three-page leaflet describing the trial. The patients sign the consent form (2 pages) at either M0 or M1 (for the sake of simplicity, referred to below as “M0” without taking the exact date, M0 or M1, into consideration). The investigator was provided with a consent questionnaire (CQ-HD), to ensure that consent was valid, and this questionnaire was completed separately by the patients and proxies (see S1 File), before the signing of the consent form. As the CQ-HD was designed as a tool to support the investigator’s explanations, its use was left to the investigator’s decision. The CQ-HD assesses how well the protocol, including its risks and procedures, is understood, providing an opportunity for feedback correction in cases of misunderstanding. It also assesses satisfaction with the information provided, and the participants’ reasons for giving consent, and their expectations of the graft. The patients were then followed up for one year. Before randomization at M12, the patients and their proxies were again asked to complete the CQ-HD, to confirm the patients’ willingness to participate in the trial and to correct any loss of information. Additional exclusion criteria are applied at this stage, to ensure there are no contraindications to surgery and no marked deterioration. Patients undergo transplantation at M13/M14 (“early graft group”) or at M33/M34 (“late graft group”).

### Outcome measures

The CQ-HD questionnaire (see S1 and S2 Files) addresses the general requirements for informed consent according to the USA Code of Federal Regulations (45 CFR 46.116(a)(1–8)). It requires yes/no or multiple-choice answers and covers three domains: 1) Understanding (maximum score 54): 17 questions assess knowledge of key aspects of the trial, such as the difference between research and care, the number of surgical procedures, graft origin, randomization, and risks; 2) Satisfaction with the information provided (maximum score 23): three questions explore the level of satisfaction with the information provided about the trial; 3) Reasons for giving consent: 3 questions examine expectations relating to the graft (5 items), the physiological role of the graft (6 items), and reasons for giving consent (10 items grouped into three domains: altruism, community influence, and personal care). Five additional questions were considered insufficiently informative for exploitation in this study.

### Statistical analyses

All participants were included in cross-sectional analyses at the time they completed the CQ-HD (M0 and/or M12), whereas only participants completing the questionnaire at both M0 and M12 were included in longitudinal analyses. We used *t* tests to compare HD patients and proxies in terms of their understanding and satisfaction with the information delivered. Analysis of variance (ANOVA) was used to test for an effect of “center” in cross-sectional and longitudinal analyses, with time (M0, M12) as a within-subject factor, and group (HD, Prox) and center (from 1 to 6) as between-subject factors. Spearman’s rank correlation analysis was applied to the patients’ demographic and clinical data, the number of patients included per center, the order of inclusions, and the patients’ CQ-HD scores. We used Chi-squared tests or Fisher’s exact tests, as appropriate, to explore expectations and reasons for giving consent. All statistical tests were two-tailed, with α = 0.05, and they were run with R software [24].

## Results

In total, 54 HD patients were included in the MIGHD study; 46 HD patients and 27 proxies completed the CQ-HD at M0, and 27 HD patients and 16 proxies completed this questionnaire at both M0 and M12. Demographic data are shown in Table 1.

**Table 1:** Demographic and clinical characteristics of the HD patients and proxies.

	M0	M12
Patients (*N*)	46	27
Age	44.3 ± 8.4 *(25–58)*	48.1 ± 7.6 *(32–59)*
Sex (% men)	68.1	74.1
Years of education	12.1 ± 3.6 *(5–20)*	12 ± 3.5 *(5–17)*
N CAG repeats (short allele)	19.3 ± 3.4 *(16–30)*	19.3 ± 3.7 *(16–30)*
N CAG repeats (long allele)	45 ± 3.7 *(40–57)*	43.9 ± 2.9 *(40–54)*
UHDRS Motor	28.1 ± 13.7 *(9–61)*	34.2 ± 13.9 *(7–61)*
UHDRS Functional	26.9 ± 1.7 *(25–32)*	27 ± 1.6 *(25–31)*
UHDRS Independence	90.5 ± 8.7 *(70–100)*	90.2 ± 7.9 *(80–100)*
UHDRS TFC	11.5 ± 1 *(10–13)*	11.3 ± 1 *(9–13)*
MDRS	131.9 ± 7 *(120–144)*	128.1 ± 7·2 *(111–144)*
Proxies (*N*)	27	16
Age	48.1 ± 11.1 *(29–71)*	51.1 ± 11.6 *(30–72)*
Sex (% men)	22.2	18.8
Spouse (*N*)	20	13
Family (*N*)	6	3
Other (*N*)	1	0

Data are numbers and means ± SD (*range*) unless otherwise indicated. N: numbers; UHDRS: Unified Huntington’s Disease Rating Scale [32]; TFC: total functional capacity [22]; MDRS: Mattis Dementia Rating Scale [23].

### Understanding

#### Cross-sectional results

The HD patients had a good understanding of the protocol at M0 (comprehension score: 39.2 ± 6.5, 72.6% correct). They largely understood the consequences of randomization (32 patients, 69.5%) and recognized the research purpose of the trial (37 patients, 80.4%). The patients recalled 5.5 ± 2 of the nine risks (60% correct) indicated on the leaflet for the trial protocol. Both comprehension and risk recall scores differed between recruiting centers (comprehension, *F*_(5, 46)_ = 4.5, *P* = 0.002; risks, *F*_(5, 46)_ = 7.8, *P*<0.001). Understanding increased with the number of patients enrolled per center (comprehension ρ = 0.3, *P* = 0.03), but not with the number of completed CQ-HD questionnaires per center. The patients’ comprehension scores correlated with their level of education (ρ = 0.36, *P* = 0.05) but not with their cognitive performance assessed with the Mattis Dementia Rating Scale [23] (ρ = 0.16, *P*>0.1). At M0, the proxies’ comprehension scores were similar to those of the patients: 39.7 ± 7.8 (73.5%) (HD vs Prox, *t* = -0.39, *P*>0.1). Comprehension subscores were similar for patients and proxies: 21 proxies (77.8%) understood the consequences of randomization (HD vs Prox, *t* = -0.71, *P*>0.1), and 22 (81.5%) understood the research purpose of the trial (HD vs Prox, *t* = -0.16, *P*>0.1). Finally, proxies recalled 6.2 ± 2.6 risks (69%) (HD vs Prox, *t* = -1.37, *P*>0.1). The comprehension scores for proxies were similar at the various centers (*F*_(5, 46)_ = 1.9, *P*>0.1).

At M12, the patients and proxies had similar comprehension scores and their understanding of randomization, the research purpose of the trial, and risks (all *Ps*>0.1) were similar, with no center effect.

#### Longitudinal results (comparison between M0 and M12)

**Fig 1:**
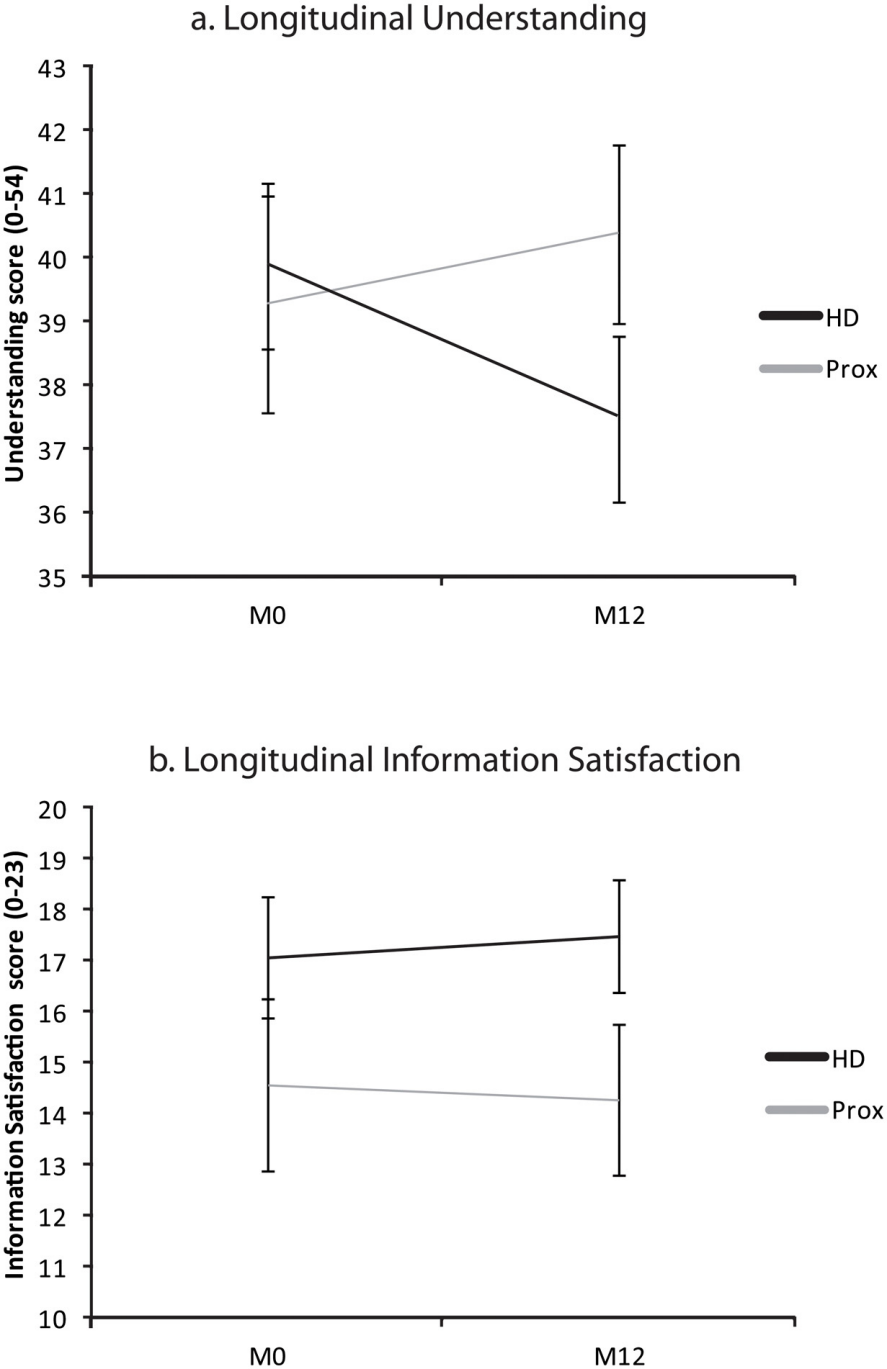
Longitudinal analysis of information processing. Huntington’s disease patients (HD) and proxies had a similar understanding of the protocol at M0. Comprehension score decreased over time in the HD patients, whereas it remained stable in their proxies (Prox). Satisfaction with the information provided remained stable in both patients and proxies. Error bars represent standard errors.

Both the comprehension and risk recall scores of HD patients decreased after one year of follow-up (comprehension score = 39.9 ± 6.8 (73.8%) vs 37.5 ± 6.7 (69.4%) respectively, paired *t* test, *t* = 2.08, *P* = 0.048; risks 5.9 ± 2.1 (65.8%) vs 4.4 ± 2.1 (48.6%) respectively, paired *t* test *t* = 3.88, *P*<0.001). By contrast, comprehension and risk recall scores remained stable in the proxies (understanding 39.3 ± 6.8 (72.8%) vs 40.4 ± 5.5 (74.8%) respectively, paired *t* test, *t* = -0.87, *P*>0.1; risks: 5.9 ± 2.8 (65.3%) vs 5.06 ± 2.2 (56.3%), paired *t* test, *t* = 1.5, *P*>0.1) (Fig 1).

### Satisfaction with information

Thirty-seven patients and 24 proxies at M0 and 24 patients and 15 proxies at M12 completed this part of the questionnaire; 20 HD patients and 15 proxies completed it at both time points.

#### Cross-sectional results

At M0, satisfaction with the information process was similar in the HD patients (16.9 ± 5, 73.5%) and their proxies (15.3 ± 6.3, 66.5%) (*t* = 1.09, *P* = 0.28), whereas, at M12, satisfaction scores were slightly higher for the patients than for their proxies (17.8 ± 4.4, 77.4% vs 14.2 ± 6, 62.2% respectively; *t* = 1.96, *P* = 0.06). The satisfaction with information score was correlated with comprehension score at both M0 (HD ρ = 0.35, *P* = 0.03; proxies ρ = 0.58, *P* = 0.003) and M12 (HD ρ = 0.62, *P* = 0.001 and proxies ρ = 0.61, *P* = 0.02).

#### Longitudinal analysis (M0–M12)

The information satisfaction score remained stable in both the HD patients (HD, M0: 17.1 ± 5.5 vs M12: 17.5 ± 4.8) and their proxies (Prox, M0: 14.6 ± 6.5 vs M12: 14.3 ± 5.8) (all *P*>0.1, paired *t* test) (Fig 1).

### Reasons for consent: expectations and motivation

Patients and proxies reported similar motivating reasons for participation at M0, and this pattern remained stable at M12 (all *Ps* >0.1). Altruism (M0: HD 88.3% vs Prox 96.4%) and personal care (M0: HD 85% vs Prox 85.7%) were key reasons for participation in both groups (Table 2). At both M0 and M12, the HD patients and proxies had similar expectations, except that the proxies were more likely than the patients to be hoping for benefits from the graft at M0 (Table 2). When asked about the physiological role of the graft at M0, two proxies (8%) and nine patients (20%) thought the graft might prevent HD progression (Fisher’s exact test, *P* = 0.3).

**Table 2:** Reasons for consenting: motivations and expectations.

	Cross-sectional M0			Longitudinal M0–M12					
	HD	Prox	*P*	HD M0	HD M12	*P*	Prox M0	Prox M12	*P*
**Motivation** (% important)									
***Influence***	**53**.**7**	**73**.**6**	ns	**56**.**4**	**60**	ns	**91**.**3**	**65**.**2**	ns
Knowing somebody	30.8	66.7	ns	42.9	50	ns	83.3	71.4	ns
Interesting	55.2	83.3	ns	55.6	68·8	ns	100	66.7	ns
Medical advice	64	69.2	ns	64.3	56·3	ns	88.9	57.1	ns
***Care***	**85**	**85**.**9**	**ns**	**85**.**6**	**92**.**8**	**ns**	**90**.**7**	**85**.**4**	ns
No alternative	69	76.5	ns	68.8	75	ns	88.9	100	ns
Better treatment	96.7	100	ns	100	100	ns	100	92.3	ns
Better care	80.8	75	ns	85.7	88.2	ns	72.7	70	ns
Only treatment	90.6	78.9	ns	89.5	100	ns	91.7	80	ns
***Altruism***	**88**.**3**	**96**.**4**	**ns**	**85**.**2**	**93**.**1**	**ns**	**97**.**1**	**100**	**ns**
Helping others	76.7	88.2	ns	70.6	87.5	ns	90.9	100	ns
For your children	93.3	100	ns	88.9	95.5	ns	100	100	ns
Scientific progress	94.1	100	ns	94.7	95	ns	100	100	ns
**Expectations** (% yes)									
Cure	26.1	18.5	ns	22.2	11.1	ns	12.5	6.3	ns
Improvement	50	63	ns	44.4	66.7	ns	68.8	87.5	ns
Stabilization	36	63	**0**.**05**	48.1	37	ns	56.3	31.3	ns
None	0	0	ns	0	3.7	ns	0	0	ns
“Don’t know”	0	0	ns	0	0	ns	0	0	ns

Patients and proxies were asked to classify each motivation as important or not important (see S1 File). ns: not significant (*p* > 0.05; Fisher’s exact test).

## Discussion

Consent to participate in research trials evaluating innovative therapies, such as cell or gene therapies, is a complex issue for populations with neurodegenerative disorders and cognitive impairment. Indeed, these studies are lengthy, complex, have an unknown risk/benefit balance, and the effects of treatment may persist beyond the end of the study. We show here, using a prospective questionnaire at inclusion (M0) and before randomization (M12), that patients with cognitive impairment can provide valid informed consent for participation in a cell transplantation trial for Huntington’s disease. Their understanding was as good as that of their proxies at M0, but had declined by M12, whereas that of their proxies had not. The proxies had motivations, expectations and satisfaction with the information delivery process similar to those of the patients, qualifying them as provisional surrogates during the course of the trial.

The HD patients and proxies had similar, good comprehension scores at inclusion, comparing favorably with some groups of healthy volunteers [25], patients without cognitive impairment [26], PD patients with mild cognitive impairment [2], and patients with dementia [13]. Despite the complex design of the MIG-HD study, the HD patients had a better understanding of the concept of randomization (70%) than that reported in previous trials for patients with arthritis (10%) [26], in vaccination trials (19%) [27], and for the parents of children with leukemia (50%) [28]. Few participants in the MIG-HD study believed the procedure would be risk-free (HD patients 2.2%, proxies 7.4%), versus 24% of 155 subjects included in 40 other clinical trials [29]. Several factors may have contributed to this high level of understanding. First, patients participating in the MIG-HD study did not have severe cognitive impairment (Mattis Dementia Rating Scale [23] ≥ 120 at M0). However, comprehension score was not correlated with cognitive performance, and even healthy participants may have a poor understanding of the trials in which they participate [25]. This calls into question the finding of a previous study, in which the authors recommended psychometric screening to identify PD patients with an impaired capacity to consent to participation in hypothetical trial scenarios [2]. Second, the information delivered in the MIG-HD study focused only on major issues, to prevent information overload [17, 25]. Third, the presence of the proxy during the information process might have enhanced the patients’ understanding, as previously shown for PD patients [30]. Fourth, the commitment of participants and investigators may be stronger in real trials (such as ours) than in simulated trials (e.g. [2]). Finally, comprehension scores were higher in centers with larger numbers of patients, suggesting that investigators with more experience are better able to adjust the information process to prevent misunderstandings.

Despite the good scores in participants’ understanding, there is still a room for improvement. Identifying patients’ or proxies’ misunderstandings is important for investigators seeking participants’ consent in future innovative research trials. First, the formulation of questions impacts on participants’ answers. For example, whereas the research aspect of the study was acknowledged by most participants (see above), only 67.4% of the patients, and 81.5% of their proxies answered at inclusion that the graft was an experimentation. Exploring the principal concerns of the trial through crossed questions might thus be useful in future studies, in order to lower the risk of therapeutic misconception. Regarding the issue of free choice, 76.1% of the patients and 74.1% of their proxies acknowledged at inclusion that participation to the protocol could be refused. Reassessment of this question after one year showed that all patients—and 81.1% of the proxies—understood it. Moreover, 76.1% of the patients and 74.1% of their proxies were aware at inclusion that withdrawal from the trial was possible at any time without explanation. It should thus be further stressed that an explanation is not required for withdrawing a trial.

Interestingly, a high level of agreement between patients and proxies was observed, suggesting that these two groups had a similar mindset concerning the trial. In addition, therapeutic misconception was infrequent: most patients (80.4%) and proxies (81.5%) recognized the research purpose of the trial and considered that the graft was unlikely to halt the disease (patients: 87.5%, and proxies: 92.6%). They did not place unreasonable hopes in the transplant procedure, although they nevertheless expected to receive better care by participating in the study. Their reasons for consenting to participation in the trial tended to be based on altruism rather than a hope for personal care, by contrast to the findings for participants in a retrospective study of the reasons for which PD patients agreed to take part in two sham-surgery controlled cell and gene therapy trials [31]. The patients and proxies reported similar levels of satisfaction with the information delivery in our study. By contrast to the findings for other studies [20, 26], their satisfaction scores were found to be correlated with their quality of understanding. This confirms that patients and proxies are similarly capable of evaluating the quality and quantity of the information delivered [20] and suggests that there was a relationship of trust between the investigators and participants. This high level of agreement between the patients and proxies further validates the role of proxies as provisional surrogates.

## Conclusion and Recommendations

Given the current growth in clinical cell and gene therapy trials, our study provides timely evidence-based data on the consent procedure. In particular, we show that Huntington’s disease patients participating in an intracerebral cell therapy trial can provide valid informed consent, although their capacity to understand the protocol may decline over time. Importantly, we found that proxies had a good, stable understanding and similar motivations to the patients, qualifying them as surrogate decision-makers. On the basis of these findings, we propose recommendations for future research trials evaluating innovative therapies in neurodegenerative disorders: 1) The patients’ understanding of the protocol and capacity to consent should be systematically assessed, through questionnaires with feedback procedures, for example; 2) Competent patients, rather than proxies, should provide consent. However, in situations in which the patient has a poor understanding or has lost competence and no alternative treatments are available (as for Huntington’s disease), proxies could be asked to endorse the consent of the patient. This approach could be refined according to the conclusions of the local ethics committee for the research concerned. 3) The identification of a person of trust [30] is crucial, as it anticipates a possible loss of patient competence during the trial or after its completion. 4) The understanding of participants should be reassessed before intervention and without advance notice, to ensure the stability of the patient’s free choice to continue participating in the trial [15]. 5) Although the present consent questionnaire did not address this issue, investigators of future innovative research trials should insist on the fact that unknown risks [4, 5] might appear in the course, or after, the research trial.

## Supporting Information

S1 File The CQ-HD questionnaire in its English version. (PDF)

S2 File The scoring method of the CQ-HD questionnaire. (PDF)
