# Supplementary material for: Longitudinal Study of Informed Consent in Innovative Therapy Research: Experience and Provisional Recommendations from a Multicenter Trial of Intracerebral Grafting
Source: PLoS One. 2015 May 26;10(5):e0128209. doi: 10.1371/journal.pone.0128209 (PMC4444138; doi:10.1371/journal.pone.0128209)
Supplement: S1 File — (PDF) [file pone.0128209.s001.pdf]

Questionnaire about informed consent in HD: CQ-HD

Procedure:

At M0: Ask the patient to read the information and the consent forms. Answer any questions and provide, even if there are no questions, a brief synthesis of important points in the protocol, then complete the questionnaire with the patient. Correct errors of interpretation concerning the protocol and re-explain obscure points to the patient. Finally, propose to the patient to sign the information and consent forms.

At M12: Fill in the questionnaire with the patient, without recalling information about the protocol. Correct errors of interpretation about the protocol and re-explain obscure points to the patient.

Patient: ………………..

Age: ………………..

Sex: M ☐ F ☐

Profession: …………………………..

---

1. Have you ever participated in an experimental study:

1.1. Yes .... ☐

1.2. No .... ☐

2. What do you think about the graft? Is it:

2.1. An experiment .... ☐

2.2. A proven treatment .... ☐

2.3. A non-proven treatment .... ☐

2.4. I don't know .... ☐

3. What do you expect from the graft:

3.1. A cure .... ☐

3.2. An improvement .... ☐

3.3. Stabilization .... ☐

3.4. Nothing special .... ☐

3.5. I don't know .... ☐

4. How many surgery procedures are you scheduled for in the protocol (free answer):

………………

5. Where do the cells come from:

5.1. From human fetuses ☐
5.2. From cell cultures ☐
5.3. From animal fetuses ☐
5.4. From stem cells ☐
5.5. From another source ☐
5.6. I don't know ☐

6. What consequences does the randomization have for you?

6.1. To be excluded from the protocol ☐
6.2. To be included in the group that will receive the graft early ☐
6.3. To be included in the group that will receive the graft late ☐
6.4. To determine which group you will belong to ☐
6.5. I don't know ☐

7. What is the duration of your participation in the study?

7.1. 1 year ☐
7.2. 13 months ☐
7.3. 32 months ☐
7.4. 52 months ☐
7.5. 72 months ☐
7.6. I don't know ☐

8. At what moment will you know when the graft will occur for you:

8.1. 12 months ☐
8.2. 13 months ☐
8.3. 24 months ☐
8.4. 32 months ☐
8.5. 52 months ☐
8.6. I don't know ☐

9. If you belong to the group that will receive the graft later, what does this represent for you:

9.1. A disaster ☐
9.2. An annoyance ☐
9.3. Nothing ☐
9.4. A good thing ☐
9.5. An intense joy ☐
9.6. I don't know ☐

10. If you belong to the group that will receive the graft earlier, what does this represent for you:

10.1. A disaster ☐
10.2. An annoyance ☐
10.3. Nothing ☐
10.4. A good thing ☐
10.5. An intense joy ☐
10.6.. I don't know ☐

11. What types of drugs are recommended in the study:

11.1. None ☐
11.2. Anticoagulants ☐
11.3. Immunosuppressants ☐
11.4. Neuroleptics ☐
11.5. Antidepressants ☐
11.6. I don't know ☐

12. Who can decide to interrupt the protocol:

12.1. You ☐
12.2. Your family ☐
12.3. Your family doctor ☐
12.4. The neurologist ☐
12.5. The sponsor ☐
12.6. Nobody ☐
12.7. I don't know ☐

13. What are the risks of the graft:

13.1. None ☐
13.2. Brain hemorrhage ☐
13.3. Bacterial infection ☐
13.4. Viral infection ☐
13.5. Hair growth ☐
13.6. Inefficacy ☐
13.7. Renal failure ☐
13.8. Cardiac failure ☐
13.9. Transplant rejection ☐
13.10. I don't know ☐

14. Is it possible that your graft may be cancelled for medical reasons despite your pre-inclusion in the protocol?

14.1. Yes ☐
14.2. No ☐

15. In your opinion, the grafts:

15.1. Can reconstruct a damaged network in the brain ☐
15.2. Can prevent the disease ☐

15.3. Have a durable effect ☐
15.4. Are effective ☐
15.5. Have a transient efficacy ☐
15.6. Are not always effective ☐

16. Does your doctor neurologist and investigator know in advance which group you will belong to:

16.1. Yes ☐
16.2. No ☐

17. The surgery will be performed:

17.1. Under general anesthesia ☐
17.2. Under local anesthesia ☐

18. What are the constraints for you:

18.1. You will not be able to participate in another study at the same time ☐
18.2. Some drugs are contraindicated ☐
18.3. You will not be allowed to go on holiday ☐
18.4. You will have to come to the hospital at dates scheduled by the neurologist ☐

19. Is randomization a problem for you:

19.1. Yes ☐
19.2. No ☐
19.3. Maybe ☐
19.4. Probably ☐
19.5. I don’t know ☐

20. What types of exams will you have during the study:

20.1. Blood tests ☐
20.2. Neurological evaluation ☐
20.3. Psychiatric evaluation ☐
20.4. Psychometric evaluation ☐
20.5. Video recordings ☐
20.6. Abdominal ultrasonography ☐
20.7. Brain imaging (MRI, PET scan) ☐
20.8. Neurophysiology ☐
20.9. I don’t know ☐

21. Who is allowed to consult the data gathered in the study:

21.1. The sponsor ☐
21.2. Your family doctor ☐
21.3. Your hospital neurologist ☐
21.4. You ☐
21.5. Your proxy ☐
21.6. Nobody ☐
21.7. I don’t know ☐

22. Do you think you have received all the necessary information about the protocol:

22.1. Yes ☐
22.2. No ☐
22.3. Maybe ☐
22.4. Probably ☐
22.5. I don’t know ☐

23. In order to really evaluate the effects of the intracerebral graft in your disease, do you think it is acceptable to propose a fake graft to some patients during fake surgery:

23.1. Yes ☐
23.2. No ☐
23.3. Maybe ☐
23.4. Probably ☐
23.5. I don’t know ☐

24. When you first heard about this protocol, were you told something like: « it is not obligatory » or « you can refuse »:

24.1. Yes ☐
24.2. No ☐

- **If yes, was this information given:**
  - 24.1.1. Orally ☐
  - 24.1.2. In writing ☐
  - 24.1.3. Both ☐
  - 24.1.4. I don’t know ☐

25. I will read to you a list of persons and would like you to say those who counted the most in providing you with information about the protocol.

	Very important	Quite important	Not very important	Not important	NA
25.1. Other patients from the hospital with whom you spoke	☐	☐	☐	☐	☐
25.2. Your hospital physician	☐	☐	☐	☐	☐
25.3. Another doctor in the medical unit	☐	☐	☐	☐	☐

25.4. Nurses	☐	☐	☐	☐	☐
25.5. Your family doctor	☐	☐	☐	☐	☐
25.6. Patients' associations	☐	☐	☐	☐	☐
25.7. Family members or relatives	☐	☐	☐	☐	☐
25.8. Others (please specify):………………………… ……………………………………………………...	☐	☐	☐	☐	☐

26. I will read three sentences to you, and would like you to tell me if, in your opinion, they are legally TRUE or FALSE.

In a protocol, from the moment when you are included:

26.1. You have to finish

- 26.1.1. True .... ☐
- 26.1.2. False .... ☐
- 26.1.3. I don't know .... ☐

26.2. You have the right to withdraw when you want

- 26.2.1. True .... ☐
- 26.2.2. False .... ☐
- 26.2.3. I don't know .... ☐

26.3. It depends, you have to discuss it with the physician

- 26.3.1. True .... ☐
- 26.3.2. False .... ☐
- 26.3.3. I don't know .... ☐

27. I will read to you a series of statements that represent reasons for participating in a protocol. I would like you to tell me those which were the most important for you.

	Very important	Quite important	Not very important	Not important	NA
27.1. Because I know other persons who did so	☐	☐	☐	☐	☐
27.2. To benefit from the best treatment	☐	☐	☐	☐	☐
27.3. Because it is a way of helping other persons	☐	☐	☐	☐	☐
27.4. To benefit from more gentle care	☐	☐	☐	☐	☐
27.5. To benefit my children	☐	☐	☐	☐	☐
27.6. For the advancement of research, of medicine	☐	☐	☐	☐	☐
27.7. Because it is the only way to benefit from the treatment	☐	☐	☐	☐	☐

27.8. Because it is interesting	☐	☐	☐	☐	☐
27.9. Because my physician thinks it is a good idea	☐	☐	☐	☐	☐
27.10. Because there is no alternative	☐	☐	☐	☐	☐

28. If you need to know something or to ask questions about the protocol, do you feel that getting additional information would be:

- 28.1. Easy .... ☐
- 28.2. Not very easy .... ☐
- 28.3. Difficult .... ☐

29. I would like now to talk about the information you received and to hear from you how it happened and what you thought about it:

- *For example, was the information you received, orally or in writing, (place a number in the corresponding gap) :*

  - ***1 Sufficient (1point)***
  - ***2 Insufficient (0 point)***
  - ***3 I don't know (0 point)***

	Orally	In Writing	NA
29.1. About the objectives of the trial			
29.2. About the risks			
29.3. About the insurance			
29.4. About the duration of the trial			
29.5. About the practical details for your organization			
29.6. About what happens if you want to withdraw			
29.7. About the agreement from the ethical committee			
29.8. About medical research law			
29.9. About the respect of anonymity			

Name of the investigator ....

SITE :................................ **DATE :** ....
