# Supplementary material for: Longitudinal Study of Informed Consent in Innovative Therapy Research: Experience and Provisional Recommendations from a Multicenter Trial of Intracerebral Grafting
Source: PLoS One. 2015 May 26;10(5):e0128209. doi: 10.1371/journal.pone.0128209 (PMC4444138; doi:10.1371/journal.pone.0128209)
Supplement: S2 File — (PDF) [file pone.0128209.s002.pdf]

Methodology for the coding procedure of the CQ-HD

Understanding Score

The Understanding score is the sum of the responses to the questions 2, 4, 5, 6, 7, 8, 11, 12, 13, 14, 16, 17, 18, 20, 21, 24, 26.

2. *The correct answer is 2.1 or 2.3. Count 1 point for each correct answer. Maximum 2 points.*

4. *The correct answer is 2. Count 1 point.*

5. *The correct answer is 5.1. Count 1 point for correct response.*

6. *The correct answer is 6.2 (1/2 point), 6.3 (1/2 point) and 6.4 (1 point). Maximum 2 points.*

7. *The correct answer is 7.4. Count 1 point for correct response.*

8. *The correct answer is 8.1. Count 1 point for correct response.*

11. *The correct answer is 11.3. The answers 11.1, 11.2, 12.4, 11.5 should not be mentioned. Count 1 point for each correct response. Maximum 5 points.*

12. *The correct answers are 12.1, 12.4, 12.5. The answers 12.2, 12.3, 12.6 should not be mentioned. Count 1 point for each correct response. Maximum 6 points.*

13. *The correct answers are 13.2, 13.4, 13.4, 13.5, 13.6, 13.7, 13.9. The answers 13.1 and 13.8 should not be mentioned. Count 1 point for each correct response. Maximum 9 points.*

14. *The correct answer is Yes. Count 1 point.*

16. *The correct answer is No. Count 1 point.*

17. *The correct answer is 17.1. Count 1 point.*

18. *The correct answers are 18.1 and 18.4. The answers 18.2 and 18.3 should not be mentioned. Maximum 4 points.*

20. *The correct answers are 20.1, 20.2, 20.3, 20.4, 20.5, 20.7, 20.8. The answer 20.6 should not be mentioned. Maximum 8 points.*

21. *The correct answers are 21.1, 21.3, 21.4. The answers 21.2, 21.5, 21.6 should not be mentioned. Maximum 8 points.*

24. *The answer 24.1 counts 1.*

26.1. *The answer 26.1.2 counts 1. The answer 26.1.1 should not be mentioned. Maximum 2 points.*

26.2. *The answer 26.2.1 counts 1. The answer 26.2.2 should not be mentioned. Maximum 2 points.*

26.3. *This question was not analyzed because it appeared unclear.*

Information satisfaction score

The Information Satisfaction score is the sum of the responses to the questions 22, 28, 29.

22. *The answer 22.1 counts 3, the answer 22.2 counts -3, the answer 22.3 counts 1, the answer 22.4 counts 2.*

28. *The answer 28.1 counts 2. The answer 28.2 counts 1. The answer 28.3 counts 0. Maximum 2 points.*

29. *Adding orally and written items amounts to the maximum score of 18.*

Reasons to consent

27. *Each participant had to response to each motivation and expectation item. Answers to motivation questions were assigned a single point and grouped into two main answers: important (very important + important) / not important (not very important, not important).*
